# Role of Orai3 in the Pathophysiology of Cancer

**DOI:** 10.3390/ijms222111426

**Published:** 2021-10-22

**Authors:** Jose Sanchez-Collado, Isaac Jardin, Jose J. López, Victor Ronco, Gines M. Salido, Charlotte Dubois, Natalia Prevarskaya, Juan A. Rosado

**Affiliations:** 1 Cell Physiology Research Group, Department of Physiology, Institute of Molecular Pathology Biomarkers, Universidad de Extremadura, 10003 Caceres, Spain; josesc@unex.es (J.S.-C.); ijp@unex.es (I.J.); victorroncodiaz4b@gmail.com (V.R.); gsalido@unex.es (G.M.S.); 2 Laboratory of Cell Physiology, INSERM U1003, Laboratory of Excellence Ion Channels Science and Therapeutics, Department of Biology, Faculty of Science and Technologiesa, University of Lille, 59650 Villeneuve d’Ascq, France; charlotte.dubois@inserm.fr (C.D.); natalia.prevarskaya@univ-lille.fr (N.P.)

**Keywords:** orai3, orai1, calcium entry, cancer

## Abstract

The mammalian exclusive Orai3 channel participates in the generation and/or modulation of two independent Ca^2+^ currents, the store-operated current, I_crac_, involving functional interactions between the stromal interaction molecules (STIM), STIM1/STIM2, and Orai1/Orai2/Orai3, as well as the store-independent arachidonic acid (AA) (or leukotriene C4)-regulated current I_a__rc_, which involves Orai1, Orai3 and STIM1. Overexpression of functional Orai3 has been described in different neoplastic cells and cancer tissue samples as compared to non-tumor cells or normal adjacent tissue. In these cells, Orai3 exhibits a cell-specific relevance in Ca^2+^ influx. In estrogen receptor-positive breast cancer cells and non-small cell lung cancer (NSCLC) cells store-operated Ca^2+^ entry (SOCE) is strongly dependent on Orai3 expression while in colorectal cancer and pancreatic adenocarcinoma cells Orai3 predominantly modulates SOCE. On the other hand, in prostate cancer cells Orai3 expression has been associated with the formation of Orai1/Orai3 heteromeric channels regulated by AA and reduction in SOCE, thus leading to enhanced proliferation. Orai3 overexpression is associated with supporting several cancer hallmarks, including cell cycle progression, proliferation, migration, and apoptosis resistance. This review summarizes the current knowledge concerning the functional role of Orai3 in the pathogenesis of cancer.

## 1. Introduction

The Orai channels are the pore-forming proteins that underlie the Ca^2+^ release-activated Ca^2+^ (CRAC) channels, the best characterized store-operated channels activated by the endoplasmic reticulum Ca^2+^ sensor STIM1 and its homolog STIM2 that mediates store-operated Ca^2+^ entry (SOCE). The Orai family of channels includes Orai1, the “classical” component of the CRAC channels, as well as two mammalian homologs, Orai2 and Orai3. The analysis of Ca^2+^ influx measurements in a genome-wide screen in patients with a form of hereditary severe combined immune deficiency (SCID) syndrome as well as in *Drosophila* S2R+ cells identified the fly gene *olf186-F* (named *Orai or CRACM1* CRAC modulator-1) as a modulator of *Drosophila* CRAC currents, and a human homolog on chromosome 12, Orai1, was found to be mutated in SCID patients, leading to the loss of CRAC channel activity [1,2,3].

Invertebrates mostly have a single *Orai* gene that is responsible for the CRAC current, although some insects, such as *Tribolium castaneum* and *Apis mellifera*, contain two Orai proteins suggesting a gene duplication in these species [4]. The crystal structure of the *Drosophila* channel revealed the hexameric architecture of the channel, where the Orai subunits arrange around a central ion pore delineated by the first transmembrane domain of the six Orai subunits [5]. In vertebrates, the *Orai* gene evolved into two branches, *Orai1* and *Orai2*, and in mammals, the *Orai3* gene made an evolutionary appearance most likely by duplication of the *Orai1* gene as it follows from the closer phylogenetic relationships with this protein [4,6].

**Figure 1:**
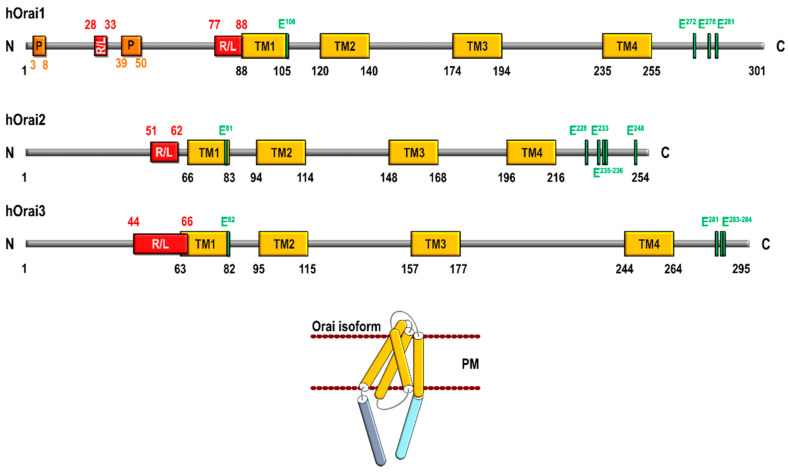
Schematic structure of human Orai1, Orai2, and Orai3 and Orai subunit architecture. R/L, arginine and lysine-rich (positively charged) region; P, proline-rich region; PM, plasma membrane; TM, transmembrane domain.

Human Orai3 is encoded by the *Orai3* gene (also known *TMEM142C*), located on chromosome 16 in the p11.2. Orai3 is a 295 amino acid protein with four transmembrane domains and N- and C-termini located in the cytosol. Orai3 shows a high percentage of homology with the Orai family members: 63.2% with Orai1 and 66.4% with Orai2 [7]. Concerning Orai3 structure, The N-terminus is composed of 65 amino acids, unlike Orai1 where this region consists of 90 amino acids and contains clusters of prolines and arginines that are absent in Orai3 [8]. Bergsmann and coworkers have reported that truncations of Orai3 N-terminal domain up to residue 51 were able to maintain STIM1-dependent activation similar to wild-type Orai3, thus suggesting that the interaction with STIM1 occurs downstream this region [9]. Orai3 N-terminus exhibits a cluster of 22 positively charged residues (H^44^-R^66^) immediately before the first transmembrane domain that is conserved in the three human Orai homologs [7] (Figure 1). Concerning this domain, the region between amino acids 42 and 62 has been reported to be essential for store-dependent activation of Orai3, in fact, the lysine residue K^60^ has been found to be crucial for the activation of store-operated currents [10]. Furthermore, deletion of Orai3 N-terminal residues between amino acids 51 and 55 has been found to increase store-operated currents. This event is unlikely mediated by the enhancement of STIM1 binding but, rather, has been attributed to an alteration of Orai3 gating with the change from fast inactivation into a potentiation phase [9]. In addition, the Orai3 N-terminus has been reported to be responsible for the selectivity of these channels for activation by arachidonic acid (AA) [11]. In the first transmembrane domain, the glutamate residue E^81^ has been identified as the channel selectivity filter (similar to E^106^ in Orai1). The second extracellular loop of Orai3 is significantly longer than that of Orai1 and Orai2 (72 residues in Orai3 vs 38 and 27 amino acids in Orai1 and Orai2, respectively) [7]. Orai3 lacks the corresponding cysteine residue C^195^ in Ora [1], which is involved in H_2_O_2_-dependent inactivation of the channel [12], this makes Orai3 resistant to oxidative stress, and reinsertion of C^195^ in Orai3 makes Orai3 redox sensitive [13]. Finally, it has been reported that the conserved glutamates, E^281^, E^283^, and E^284^, form a motif that is responsible for fast Ca^2+^-dependent inactivation of Orai3, as mutating these glutamates to alanines abrogated the fast Ca^2+^-dependent inactivation [14].

STIM1-operated Orai1 currents are 2–3 fold greater than that of its homologs Orai2 and Orai3 [15], a phenomenon that might be attributed to different biophysical properties. First, fast Ca^2+^-dependent inactivation (FCDI) of STIM1-mediated currents is about three times more pronounced in Orai3 than in Orai1, and, in turn, Orai1 exhibits an FCDI that is about half as pronounced as in Orai2 [16,17]. Second, STIM1-operated Orai1 currents exhibit a reactivation phase, after the FCDI, upon the application of a hyperpolarizing voltage step. This feature is exclusive of Orai1 channels, whereas Orai2 and Orai3 currents do not exhibit a reactivation phase [16,18,19]. Third, the half-maximal channel activation time is significantly greater for Orai3 (approx. 63 s) than for Orai1 or Orai2 (35 and 21 s, respectively) [16]. The mentioned differences are likely due to certain differences in the cytosolic regions of the Orai isoforms. It has been reported that the Orai1 specific proline/arginine-rich domains located in the N-terminus are essential for current reactivation, while the second, cytosolic, loop seems to be involved in fast and slow gating processes [19]. Finally, FCDI of Orai2 and Orai3 has been reported to be largely dependent on three conserved C-terminal glutamates, E^233^, E^235^, and E^236^ of Orai2 and E^281^, E^283^, and E^284^ of Orai3 [14]; however, a different mechanism for Ca^2+^-dependent inactivation has been reported for Orai1 associated to the interaction with and activation of adenylyl cyclase 8, which, in turn, leads to protein kinase A-dependent phosphorylation of Orai1 at S^34^ [20].

Orai3 participates in the generation of two different Ca^2+^ currents, including the store-operated current: I_crac_ and the AA (or leukotriene C4)-operated current I_arc_. Native CRAC channels have been reported to be formed by a hexameric assembly of Orai1/2/3 subunits, where Orai2 and Orai3 modulate Ca^2+^ entry through CRAC channels in order to match the magnitude of Ca^2+^ signals to agonist stimulation [21,22]. In addition to its participation in the store-operated CRAC channels, Orai3 is an essential component of the store-independent, AA-regulated Ca^2+^ (ARC) channels [23]. Functional ARC channels consist of a heteropentameric assembly of three Orai1 subunits and two Orai3 subunits and show differential characteristics with CRAC channels, including independence of intracellular Ca^2+^ store depletion, activation by low concentrations of AA, and complete requirement for the minor pool of STIM1 that constitutively resides in the plasma membrane [24]. As mentioned above, the N-terminus of Orai3, more specifically, the region between amino acids 1–38, which is remarkably different from the corresponding residues in Orai1, is critical for the selective activation of ARC channels by AA [11]. Orai3 has been also involved in the generation of the store-independent current activated by leukotriene C4; however, the comparison of the currents generated by AA and leukotriene C4 in different cell types shows that they are indistinguishable and non-additive, thus suggesting that both currents are mediated by the same channel [25]. It is noteworthy that high (20–50 µM [26]) concentrations of 2-aminoethoxydiphenyl borate (2-APB), initially identified as a non-selective inhibitor of the inositol 1, 4, 5-trisphosphate (IP_3_) receptors and transient receptor potential (TRP) channels [27,28] is able to activate Orai3 currents, altering the ion selectivity of the channel by increasing Orai3 channel minimum pore size from about 3.8 Å to more than 5.34 Å. The effect of 2-APB on Orai3 activation has been reported to be dependent on the E^165^ residue located in the third transmembrane domain, as the Orai3-E165Q mutant resulted in permeation properties similar to that observed by 2-APB stimulation of Orai3 and almost completely abolished the stimulatory effect of 2-APB [29].

Overexpression of functional Orai3 has been reported in several neoplastic cells where this channel has been associated to the development or support of several cellular activities, thus leading to cancer progression.

## 2. Orai3 in Breast Cancer

**Figure 2:**
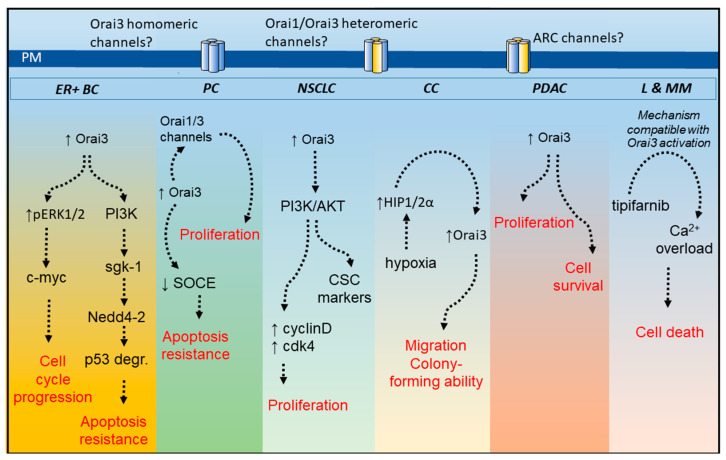
Overview of Orai3 functional role in cancer cells. Orai3 expression has been reported to be up-regulated in different cancer cell types, including those from estrogen receptor-positive breast cancer (ER + BC), prostate cancer (PC), non-small cell lung cancer (NSCLC) cells, colorectal cancer (CC), pancreatic ductal adenocarcinoma (PDAC) as well as tipifarnib-sensitive acute myeloid leukemia and multiple myeloma (L and MM). Except in leukemia and multiple myeloma cells, Orai3 plays a relevant role in the development or maintenance of different cancer hallmarks, such as cell cycle progression and proliferation, apoptosis resistance and survival and migration and colony formation. In tipifarnib-sensitive leukemia and multiple myeloma cells Orai3-mediated Ca^2+^ influx is activated by tipifarnib leading to Ca^2+^ overload and loss of cell viability. PI3K, phosphatidylinositol 3-kinase; ARC channels, arachidonic acid-regulated calcium channels; ERK, extracellular signal-regulated kinase; sgk-1, serine/threonine-protein kinase-1; p53 degr., p53 degradation; cdk-4, cyclin-dependent kinase-4; CSC, cancer stem cells; HIP1/2α, hypoxia inducible factor 1/2α; ↑ means overexpression or activation and ↓ means inhibition.

Breast cancer is among the most common cancer types and one of the major causes of cancer death in women. From the point of view of the gene expression profile, breast cancer is a heterogeneous disease that can be grouped into different subtypes, including luminal A, luminal B, HER2, and basal or triple-negative subtypes [30]. Orai3 has been reported to be highly expressed in the estrogen receptor-positive (ER+) breast cancer cell lines MCF7, ZR751, T47D, and HCC1500 [31] as well as in luminal breast cancer tissues as compared to non-tumoral breast epithelial cells and adjacent non-cancerous tissues [32,33]. Orai3 expression in MCF7 breast cancer cells has been reported to be dependent on ERα since ERα knockdown significantly reduced Orai3 expression in these cells. Conversely, it has been reported that attenuation of Orai3 expression significantly inhibits ERα+ cell tumorigenesis in immunodeficient mice [34]. In ER+ breast cancer cells, Orai3 expression plays a relevant role in the maintenance of a variety of cancer hallmarks, including cell cycle progression, cell proliferation, migration, and cell survival [31,32,35], thus unveiling the potential role of Orai3 as a therapeutic target for antitumoral strategies. Studies in the ER+ breast cancer cell line MCF7 have provided evidence supporting that the c-myc proto-oncogene is modulated by Ca^2+^ influx through Orai3 by a mechanism including the MAP kinase pathway as Orai3 knockdown results in a reduction in the expression and activity of c-myc, decreased pERK1/2 levels and cell-cycle arrest in G1 phase [36]. In addition, to its role in cell proliferation and migration in ER+ breast cancer cells, Orai3 has been reported to be involved in breast cancer cell resistance to chemotherapeutic drugs [37]. Ca^2+^ influx via Orai3 confers apoptosis resistance by modulating p53 protein expression through the activation of the PI3K/Sgk-1 signaling pathway, which, in turn, activates the ubiquitin ligase Nedd4-2 and results in p53 ubiquitinylation and subsequent degradation [37] (Figure 2).

In ER+ breast cancer cell lines, SOCE has been reported to be strongly dependent on Orai3 expression [31], while Orai1 knockdown in these cells was predominantly without effect, in contrast to the observations in triple-negative breast cancer cell lines [38,39]. Orai3-mediated SOCE in ER+ breast cancer cells has been shown to be modulated by the TRP channel, TRPC6, as TRPC6 knockdown or transient expression of a dominant negative TRPC6 mutant resulted in attenuation of the plasma membrane expression of Orai3 and well as SOCE inhibition in MCF7 cells [35]. As a result of its role in SOCE, knockdown of Orai3 alone or in combination with STIM2 and TRPC6 leads to impairment of resting cytosolic as well as endoplasmic reticulum Ca^2+^ concentration in MCF7 cells [40].

The potential mechanisms underlying Orai3 up-regulation in ER+ breast cancer cells remain unclear. Recent studies have reported that hypoxia, but not the epidermal growth factor (EGF), another EMT (epithelial–mesenchymal transition) inducer, is able to increase Orai3 expression in the triple-negative breast cancer cells MDA-MB-468, HCC1569 and MDA-MB-231 as well as in the EMT breast cancer cell line PMC42LA, while the other Orai proteins, Orai1 and Orai2, were not up-regulated. Nevertheless, under these conditions, Orai3 knockdown failed to attenuate SOCE, thus suggesting that an alternative mechanism might underlie Orai3 up-regulation and function in ER+ breast cancer cells [33]. Functional studies in ER+ breast cancer cells combined with bioinformatic analysis have demonstrated that miR18a and miR18b positively regulate Orai3 expression and function while miR34a represses Orai3 expression by inducing translational block [41].

Orai3 has also been reported to be expressed in triple-negative MDA-MB-231 breast cancer cells. However, in these cells, Orai1 is the predominant constituent of the channels mediating SOCE [31,42] which challenged the relevance of Orai3 in Ca^2+^ homeostasis. Interestingly, while these cells express all the components of the ARC channels, i.e., Orai1, Orai3, and STIM1, stimulation with AA was unable to induce Ca^2+^ influx but, rather, attenuated the ability of MDA-MB-231 cells to migrate and proliferate by inducing disruption of the mitochondrial membrane potential and the activation of apoptosis [43]. Nevertheless, cell stimulation with 2-APB revealed the presence of functional Orai3 channels in MDA-MB-231 cells, and Orai3 knockdown unveiled that Orai3 protects the cells against the harmful effects of AA [43].

## 3. Orai3 in Prostate Cancer

According to the World Health Organization 2021, prostate cancer (PCa) is the most common non-cutaneous human malignancy and the third most lethal tumor among men, with the highest incidence in industrialized countries. The incidence of prostate carcinoma, over half a million new cases every year, increases proportionally to the increase in life expectancy. Orai3 (as Orai1 and Orai2) is expressed in normal and cancerous epithelial prostatic cells. It has been shown that the remodeling of channel-forming Orai proteins determines an oncogenic switch in prostate cancer (PCa) [44]. This study showed that the relative expression level of the Orai3 transcript is strongly increased in PCa samples compared to paired-match non-cancerous tissue. Authors have shown that the endogenous association of Orai1 and Orai3, leads to the formation of store-independent ARC channels activated by AA. Interestingly, neither co-immunoprecipitation nor biotinylation assays have allowed the authors to confirm the implication of STIM1 in the mechanism of AA-mediated Ca^2+^ entry in PCa cells. These results suggest that in PCa models, the mechanism of AA-mediated Ca^2+^ entry differs from the classical ARC channel previously described [24].

The activation of Orai1/Orai3 channels by AA allows an increase in cytosolic calcium concentration that leads to the nuclear translocation of NFAT, a Ca^2+^/calcineurin-dependent transcription factor involved in cell proliferation. Moreover, the silencing of Orai3 decreases proliferation in vitro and in vivo using a PC3-xenograft mouse model, via a decreased cyclin D1 expression, a key controller of the G1/S phase cell cycle. On the opposite, overexpression of Orai3 promotes proliferation both in vitro and in vivo. These results show the implication of Orai3 during PCa cells proliferation [44].

By mimicking Orai protein remodeling observed in primary tumors (Orai1:Orai3 1:3 ratio), the authors demonstrated that enhanced Orai3 expression favors its heteromerization with Orai1 which impedes the formation of homomeric Orai1 channels. Thus, the increase of Orai1/Orai3 channels reduces the number of functional homomeric Orai1 channels, which are important in supporting susceptibility to apoptosis [44].

In addition to the constitutive effect of this remodeling, the treatment with AA also reduces the formation of Orai1 channels. Indeed, authors have shown in vitro and ex vivo using freshly isolated primary human PCa cells (obtained after radical prostatectomy) that pretreatment with AA decreases SOCE [44]. Different studies have shown that the AA signaling pathway (phospholipase, cyclooxygenase, lipoxygenase, and epoxygenase enzymes) has a major role in the development and progression of several solid cancer among PCa [45,46]. In their study, Dubois and coworkers provide insights concerning the role of AA in PCa oncogenesis by establishing a direct link between AA metabolism and PCa cells proliferation via the involvement of AA-regulated Ca^2+^ entry [44]. Recent studies have demonstrated the crucial role of tumoral parasympathetic cholinergic fibers which activate muscarinic pathways in stromal participating in PCa progression [47]. Furthermore, the study by Dubois and colleagues has demonstrated that Orai1/Orai3 heteromers promote proliferation following stimulation of M3 muscarinic receptors (M3-AChR) [44], thus further investigations are needed to evaluate if PCa progression mediated by parasympathetic cholinergic fibers is at least partly due to Orai1/Orai3 heteromers.

## 4. Orai3 in Lung Cancer

There are different types of primary lung cancer that can be grouped into two categories: small cell lung cancer (SCLC) or neuroendocrine tumors, which accounts for approximately 15–20% of lung cancer diagnosed, and non-small cell lung cancer (NSCLC), which can be grouped into adenocarcinoma, squamous cell carcinoma and large cell carcinoma [48]. Most studies concerning the expression and functional role of Orai3 in lung cancer have been performed by the Ouadid-Ahidouch group, who initially analyzed Orai3 expression by immunohistochemistry in samples of lung tumors and adjacent tissue from the same patients reporting that ~66% of the tumoral tissue exhibited a strong Orai3 expression as compared to non-tumoral tissue samples [49]. Interestingly, Orai3 staining was found to be more elevated in higher tumor grades [49]. Further analysis in a larger cohort of lung cancer patients confirmed that Orai3 is overexpressed in tumoral samples as compared to adjacent non-tumoral tissue but revealed that the expression of Orai3 does not correlate with EGF receptor mutation, KRAS mutation, or the TNM stage (classification of malignant tumors based on the size, lymph nodes involved and metastasis) [50]. This analysis also showed that overall survival was greater in individuals with a lower Orai3 expression, thus suggesting that Orai3 is a prognostic factor for lung cancer outcome [50].

Studies performed in two NSCLC cell lines, NCI-H23 and NCI-H460, have revealed that SOCE is strongly dependent on Orai3 expression while silencing of Orai1 or Orai2 expression failed to impair SOCE [49]. In these cells, Orai3 knockdown attenuates cell proliferation and induces cell cycle arrest at the G1 phase, an event that is likely mediated by inhibition of AKT activation, thus decreasing the expression of cyclin D1 and Cdk4 [49] (see Figure 2).

A recent study has reported that Orai3 induces cisplatin resistance in NSCLC cells by enriching the population of cancer stem cells [51]. Unlike Orai1, Orai3 expression is significantly enhanced in bronchial biopsies after treatment with cisplatin, which shifts the Orai1:Orai3 expression ratio in favor of Orai3, enhances SOCE, and is associated with the expression of cancer stem cells markers, Nanog and SOX-2, most likely mediated by a mechanism dependent on the activation of the PI3K/AKT pathway [51]. Interestingly, this study also reveals that the activation of the PI3K/AKT pathway is essential for the increase in Orai3 expression after treatment with cisplatin [51], which suggest a positive feedback mechanism in the regulation of Orai3 expression and PI3K/AKT activation upon treatment with cisplatin and reveals that Orai3 might be a useful target to monitor the lung cancer outcome upon the treatment with chemotherapeutic drugs.

## 5. Expression and Functional Role of Orai3 in Other Types of Cancer

In addition to the above-mentioned pathophysiological roles of Orai3, this channel has been involved in the development or maintenance of different cancer hallmarks in a variety of tumors, including myeloid leukemia, colorectal cancer, and pancreatic adenocarcinoma (Figure 2).

Studies performed in the acute myeloid leukemia cell line U937 and the multiple myeloma cell line 8226 have provided evidence for a role of Orai3 in tipifarnib-induced altered Ca^2+^ homeostasis [52]. Treatment of the tipifarnib-sensitive cell lines U937 and 8226 with tipifarnib, a farnesyltransferase inhibitor that prevents farnesylation and, thus, activation, of the Ras protein [53], results in a rise in cytosolic Ca^2+^ concentration due to Ca^2+^ entry through plasma membrane channels by a mechanism compatible with Orai3 activation [52]. Tipifarnib-evoked Ca^2+^ entry leads to Ca^2+^ overload and loss of membrane integrity. Interestingly, in the tipifarnib-resistant cell line 8226/R5, the expression of Orai3 is significantly smaller than in the parental, tipifarnib-sensitive, cell line 8226, thus suggesting that Orai3 plays a relevant role in the pharmacological effects of tipifarnib [52].

In the human colorectal cancer cell line HT29, the expression of Orai3, as well as Orai1, Orai2, STIM1, and TRPC1 has been shown to be enhanced as compared to the normal human colon mucosal epithelial cell line NCM460, whereas STIM2 expression was downregulated in cancer cells [54]. In these cells, Orai2 and Orai3 seem to modulate Ca^2+^ entry through CRAC channels as the knockdown of these channels increases SOCE [54]. Further analysis in tumor samples from murine colon cancer xenograft models and patients have reported that hypoxia increases HIF-1/2α expression concomitantly with enhanced Orai1 and Orai3 expression and SOCE. Unlike Orai1, Orai3 expression was found to be dependent on HIF-1/2α expression level or activation. The colony-forming ability and cell migration capacity of tumor cells were impaired after treatment with siHIF-1α and siHIF-2α, as well as siOrai3, thus indicating that HIF-1/2α promote Orai3 up-regulation, which, in turn, supports colon cancer progression [55].

Finally, a recent study has revealed that Orai3 plays an essential role in cell proliferation in pancreatic ductal adenocarcinoma [56]. Orai3 was found to be overexpressed in pancreatic tumor samples from patients as well as in a variety of pancreatic adenocarcinoma cell lines (BxPC3, Capan1, MiaPaCa2, and Panc1) and a cell line derived from metastatic ascites (ASPC1) as compared to normal tissue and the non-tumoral human pancreatic duct epithelial H6C7 cell line. Orai3 channel function has also been found to be remodeled in cancer cells, as Orai3 knockdown enhances SOCE in pancreatic cancer cells whereas attenuates Ca^2+^ influx in non-tumoral cells. Interestingly, Orai3 silencing was associated with a decrease in cell proliferation, via cell cycle arrest in the G2/M-phase. In addition, further studies in tumor MiaPaCa2 cells indicate that Orai3 knockdown impairs cell survival and induces mitotic catastrophe and apoptosis, as well as inhibits tumor growth and induces apoptosis in vivo using nude mice bearing MiaPaCa2-derived xenografts. These findings strongly suggest that Orai3 is a promising therapeutic target for pancreatic adenocarcinoma [56].

In other cancer cells, such as the oral cancerous SAS cells, the expression of Orai3 is reduced in comparison with non-cancerous HaCaT cells, although the potential relevance of this finding has not been further explored. In cancer cells, Orai1 and Orai2 expression is upregulated and modulates cell migration by regulation of the AKT/mTOR/NF-κB signaling pathway [57].

## 6. Conclusions

To summarize, Orai3 expression and function have been reported to be remodeled in different cancer cells. In the investigated cells, Orai3 is predominantly overexpressed as compared to non-tumor cells but its role in SOCE/Ca^2+^ influx differs in the different cancer cell types. In most cell types, knockdown of Orai2 and Orai3 does not abolish SOCE while knockdown of Orai1 does [6]. The functional role of Orai2 and Orai3 in SOCE is mostly associated with the modulation of the magnitude of Ca^2+^ influx by heteromultimerization with Orai1 subunits into the CRAC channels [22]. Among the exceptions are ER+ breast cancer cells and NSCLC cells, where SOCE is strongly dependent on the Orai3 expression, while knockdown of Orai1 or Orai2 has a minor effect if any, meaning that Orai3 plays a predominant role in SOCE in these cells. This unprecedented functional role of Orai3 has a significant effect on the biology of these cancer cells, where Orai3 plays a relevant role in cell cycle progression, cell proliferation, and apoptosis resistance (Figure 2). In prostate cancer, Orai3 upregulation is associated with SOCE attenuation and formation of Orai1/Orai3 heteromers regulated by AA. This remodeling results in enhanced proliferation and apoptosis resistance (Figure 2). In colorectal cancer and pancreatic adenocarcinoma cells, the overall functional role of Orai3 in SOCE is the classically identified modulation of SOCE, as Orai3 knockdown in these cells has been found to increase SOCE. In addition, Orai3 has also been associated with different cancer hallmarks, such as cell migration, proliferation, colony formation, or cell survival in colorectal cancer and pancreatic adenocarcinoma cells (Figure 2). An interesting finding has been reported in tipifarnib-sensitive leukemia and multiple myeloma cell lines where Orai3 is responsible for tipifarnib-mediated Ca^2+^ overload, which, in turn, plays an essential role in the cytotoxic effects of tipifarnib (Figure 2). At present, the architecture of the functional Orai3-forming channels in cancer cells is unclear but, altogether, there is a body of evidence supporting a relevant role for Orai3 in tumorigenesis, which strongly suggests that Orai3 is a potential target both as a biomarker of prognosis and as an objective for the development of anticancer therapeutic strategies. A variety of synthetic modulators of the Orai channels have been identified, including BTP2, GSK-7975A, synta66, 2-APB, or IA65, with the Orai isoforms exhibiting different sensitivity and pharmacological profiles to these agents. BTP2 and GSK-7975A are well-known inhibitors of Orai3, although they are less robust inhibitors of the channel than trivalent cations. On the other hand, synta66 is ineffective on Orai3 function while 2-APB (at high micromolar concentrations) is a well-known activator of Orai3 and IA65 is a modest Orai3 activator (26). The potential role of the Orai3 modulators in cancer therapy deserves further study.

## Data Availability

The data presented in this study are available on request from the corresponding author.
